# Supplementary material for: Glycan heterogeneity as a cause of the persistent fraction in HIV-1 neutralization
Source: PLoS Pathog. 2023 Oct 30;19(10):e1011601. doi: 10.1371/journal.ppat.1011601 (PMC10635575; doi:10.1371/journal.ppat.1011601)
Supplement: S1 Table — (PDF) [file ppat.1011601.s001.pdf]

S1 Table. Single-cycle kinetics (SCK)

Validation of Langmuir modeling [a]

**Fab**	**Affinity purification**	***T (k_{on})***	***T (k_{off})***	χ^2	k_t (RU/Ms)	***T (k_t)***	***U***
3BNC117	2G12 (n=2)	$1.5 \cdot 10^3$ ± 50	$7.7 \cdot 10^2$ ± 40	0.10 ± $3.5 \cdot 10^{-4}$	$1.0 \cdot 10^{21}$ ± $1.0 \cdot 10^{21}$	$1.1 \cdot 10^{-2}$ ± $1.1 \cdot 10^{-2}$	1.0 ± 0

[a] Tabulated values are means ± S.E.M of ‘n’ independent replicates. The data complement **Figure 10**.

Validation of Heterogeneous-ligand modeling [a]

**Fab**	**Affinity purification**	***T (k_{on1})***	***T (k_{off1})***	***T (k_{on2})***	***T (k_{off2})***	χ^2	k_t (RU/Ms)	***T (k_t)***
PGT151	2G12 (n=2)	$3.4 \cdot 10^2$ ± $1.9 \cdot 10^2$	$2.9 \cdot 10^2$ ± 70	$3.0 \cdot 10^2$ ± $1.3 \cdot 10^2$	$2.7 \cdot 10^2$ ± 0	$5.9 \cdot 10^{-2}$ ± $1.4 \cdot 10^{-2}$	$2.6 \cdot 10^{17}$ ± $1.1 \cdot 10^{17}$	$4.4 \cdot 10^{-5}$ ± $2.4 \cdot 10^{-5}$
	PGT151 (n=4)	$3.1 \cdot 10^2$ ± $1.2 \cdot 10^2$	$2.4 \cdot 10^2$ ± 43	$2.9 \cdot 10^2$ ± $1.1 \cdot 10^2$	$2.3 \cdot 10^2$ ± 45	0.18 ± $6.0 \cdot 10^{-3}$	$3.6 \cdot 10^{19}$ ± $3.5 \cdot 10^{19}$	$2.1 \cdot 10^{-4}$ ± $1.7 \cdot 10^{-4}$

[a] Tabulated values are means ± S.E.M of ‘n’ independent replicates. The data complement **Figure 11**.
