# Supplementary material for: Glycan heterogeneity as a cause of the persistent fraction in HIV-1 neutralization
Source: PLoS Pathog. 2023 Oct 30;19(10):e1011601. doi: 10.1371/journal.ppat.1011601 (PMC10635575; doi:10.1371/journal.ppat.1011601)
Supplement: S2 Table — (PDF) [file ppat.1011601.s002.pdf]

S2 Table. Multi-cycle Kinetics (MCK)

Validation of Langmuir modeling [a]

**Fab**	**Affinity purification**	***T* (k_{on})**	***T* (k_{off})**	χ^2	**k_t** (RU/Ms)	***T* (k_t)**	***U***
3BNC117	2G12 (n=2)	$2.8 \cdot 10^3$ ± 50	$3.0 \cdot 10^2$ ± 20	$6.0 \cdot 10^{-2}$ ± $5.0 \cdot 10^{-4}$	$6.2 \cdot 10^{20}$ ± $6.2 \cdot 10^{20}$	$4.7 \cdot 10^{-2}$ ± $4.7 \cdot 10^{-2}$	3.0 ± 0

[a] Tabulated values are means ± S.E.M of 'n' independent replicates. The data complement **SI Figure 4**.

Validation of Heterogeneous-ligand modeling [a]

**Fab**	**Affinity purification**	***T* (k_{on1})**	***T* (k_{off1})**	***T* (k_{on2})**	***T* (k_{off2})**	χ^2	**k_t** (RU/Ms)	***T* (k_t)**
PGT151	2G12 (n=2)	$7.1 \cdot 10^2$ ± 5.0	$1.0 \cdot 10^2$ ± 70	$2.8 \cdot 10^2$ ± 65	$3.4 \cdot 10^2$ ± 65	$2.3 \cdot 10^{-2}$ ± $4.1 \cdot 10^{-3}$	$3.9 \cdot 10^{21}$ ± $3.5 \cdot 10^{20}$	$8.1 \cdot 10^{-4}$ ± $4.0 \cdot 10^{-4}$

[a] Tabulated values are means ± S.E.M of 'n' independent replicates. The data complement **SI Figure 4**.
