# Supplementary material for: Glycan heterogeneity as a cause of the persistent fraction in HIV-1 neutralization
Source: PLoS Pathog. 2023 Oct 30;19(10):e1011601. doi: 10.1371/journal.ppat.1011601 (PMC10635575; doi:10.1371/journal.ppat.1011601)
Supplement: S3 Table — (PDF) [file ppat.1011601.s003.pdf]

S3 Table. Cryo-EM data collection, processing, and validation

**Map**	**CZA97.012 SOSIP + 3BNC117 Fab**
EMDB	EMD-40088
**Data collection**	
Microscope	Thermo Fisher Titan Krios
Voltage (kV)	300
Detector	Gatan K2 Summit
Recording mode	Counting
Nominal magnification	22,500
Movie micrograph pixelsize (Å)	1.31
Dose rate (e^-/[(camera pixel)*s])	10.0
Number of frames per movie micrograph	50
Frame exposure time (ms)	200
Movie micrograph exposure time (s)	10
Total dose (e^-/Å^2)	58
Defocus range (µm)	-1.0 to -2.7
**EM data processing**	
Number of movie micrographs	1,142
Number of molecular projection images in map	79,261
Symmetry	C3
Map resolution (FSC 0.143; Å)	3.4
Map sharpening B-factor (Å^2)	-126
**Structure Building and Validation**	
Number of atoms in deposited model	
SOSIP gp120	10,707
SOSIP gp41	2,925
Fab Fv	5,349
glycans	1,677
MolProbity score	1.00
Clashscore	1.63
Map correlation coefficient	0.83
d FSC model (0.5)	3.6
EMRinger score	2.82
RMSD from ideal	
Bond length (Å)	0.006
Bond angles (˚)	0.946
Ramachandran plot	
Favored (%)	97.57
Allowed (%)	2.43
Outliers (%)	0.00
Side chain rotamer outliers (%)	0.29
Cβ outliers (%)	0.00
PDB	8gje
