# Supplementary material for: Glycan heterogeneity as a cause of the persistent fraction in HIV-1 neutralization
Source: PLoS Pathog. 2023 Oct 30;19(10):e1011601. doi: 10.1371/journal.ppat.1011601 (PMC10635575; doi:10.1371/journal.ppat.1011601)
Supplement: S1 Fig — (PDF) [file ppat.1011601.s004.pdf]

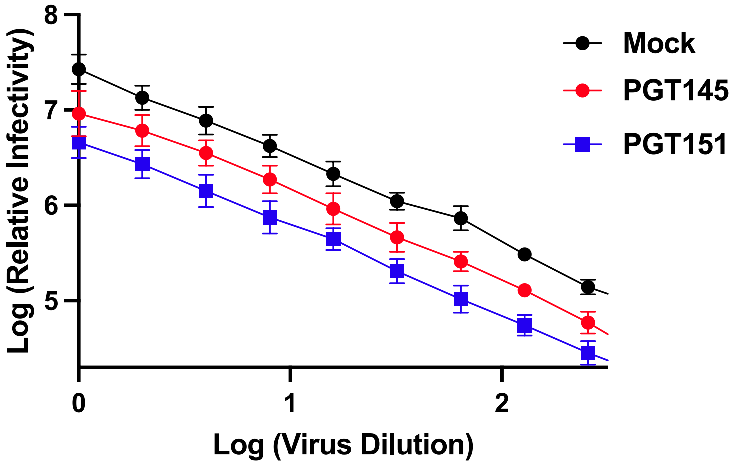

S1 Figure. The residual infectivity in neutralization of PV at varied dose. The $\log_{10}$ of the relative remaining infectivity after neutralization by bNAb at a fixed concentration (50 μg/ml) is plotted as a function of the $\log_{10}$ of varied inoculum-dilution factor.
