# Supplementary material for: Glycan heterogeneity as a cause of the persistent fraction in HIV-1 neutralization
Source: PLoS Pathog. 2023 Oct 30;19(10):e1011601. doi: 10.1371/journal.ppat.1011601 (PMC10635575; doi:10.1371/journal.ppat.1011601)
Supplement: S2 Fig — (PDF) [file ppat.1011601.s005.pdf]

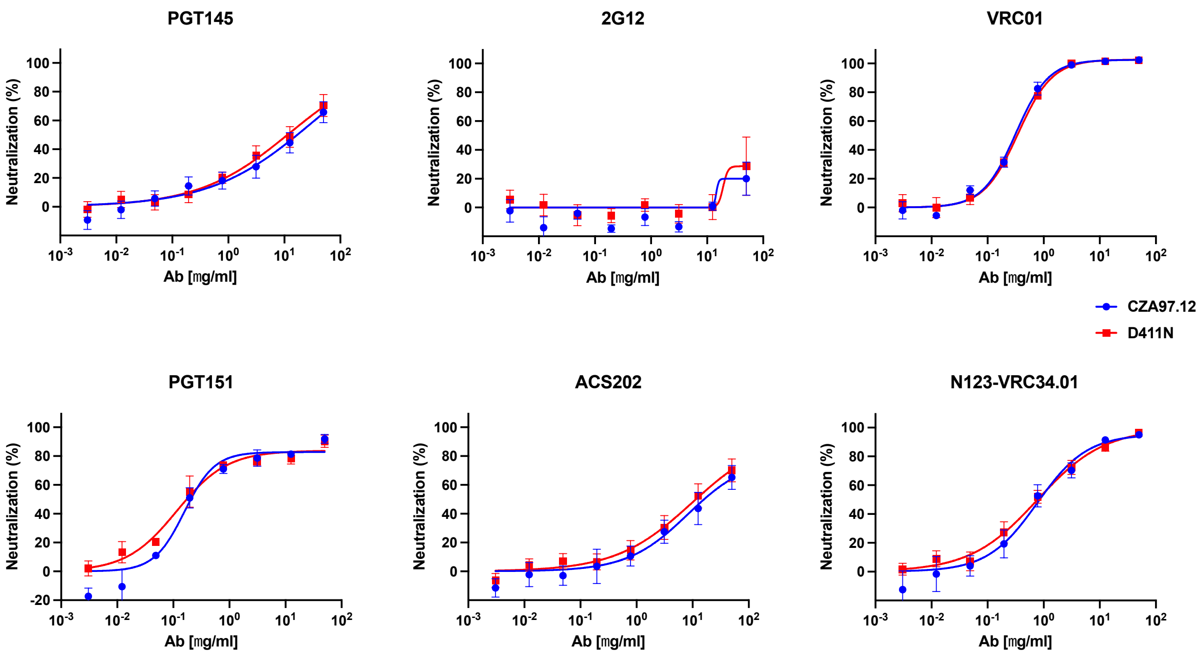

S2 Figure. Neutralization of CZA97.012 parental and D411N-mutant PV. Neutralization of the respective PV (see legend) by the indicated bNAbs is shown. The diagrams show a sigmoid curve fitted to % neutralization as a function of bNAb concentration (µg/ml). The data points are means of 2-9 replicates ± s.e.m.
