# Supplementary material for: Glycan heterogeneity as a cause of the persistent fraction in HIV-1 neutralization
Source: PLoS Pathog. 2023 Oct 30;19(10):e1011601. doi: 10.1371/journal.ppat.1011601 (PMC10635575; doi:10.1371/journal.ppat.1011601)
Supplement: S3 Fig — (PDF) [file ppat.1011601.s006.pdf]

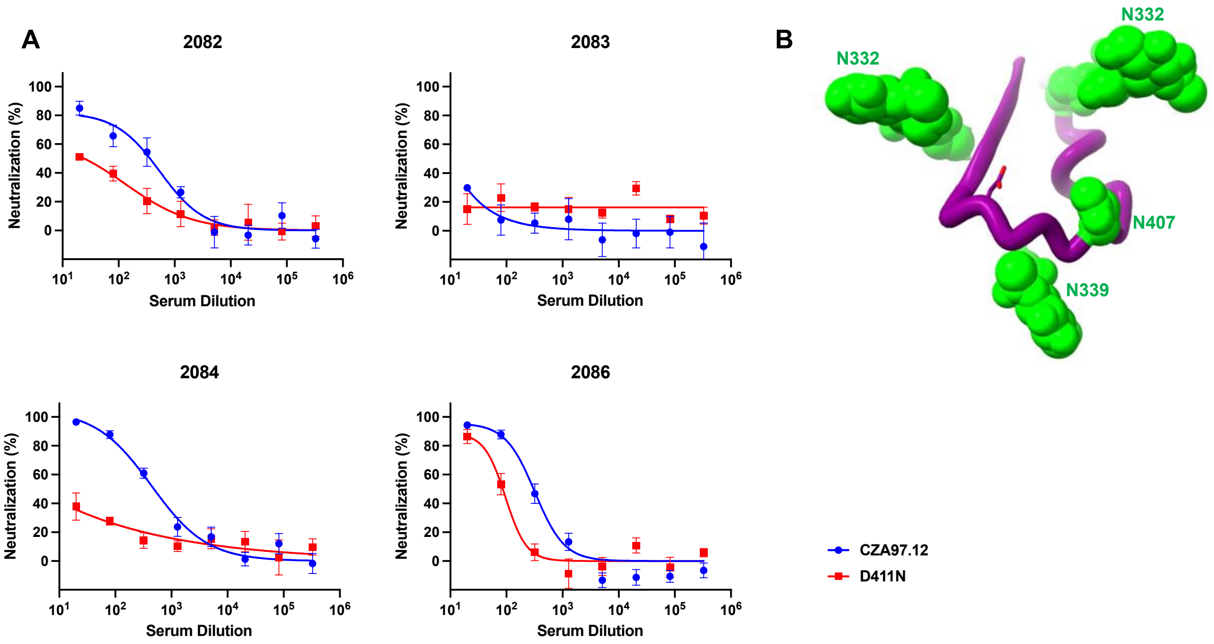

S3 Figure. The mapping of autologous NAb epitopes in sera from rabbits immunized with CZA97.012 SOSIP.664. A. The immunization sequence (70) and the D411N mutant have been described (69). Neutralization of CZA97.012 parental and D411N-mutant PV by rabbit sera was analyzed. The diagrams show a sigmoid curve fitted to % neutralization as a function of the serum dilution factor. The data points are means of 5-11 replicates ± s.e.m. **B.** A cryo-EM model of the CZA97.012 SOSIP.664 V4-β19 transition (backbone in purple) is shown with N-linked glycans near D411 (side-chain as red stick) depicted with carbohydrate residues as green spheres.
