# Supplementary material for: Glycan heterogeneity as a cause of the persistent fraction in HIV-1 neutralization
Source: PLoS Pathog. 2023 Oct 30;19(10):e1011601. doi: 10.1371/journal.ppat.1011601 (PMC10635575; doi:10.1371/journal.ppat.1011601)
Supplement: S4 Fig — (PDF) [file ppat.1011601.s007.pdf]

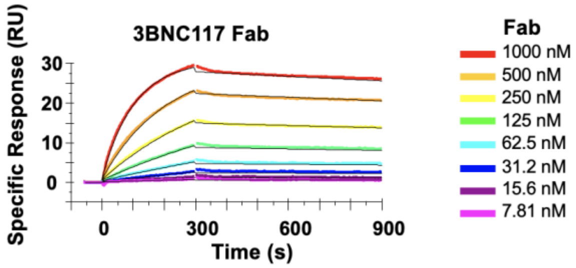

Langmuir modeling of 3BNC117 Fab binding to CZA97.012 SOSIP.664

Fab	Affinity purification	k_{on} (1/Ms)	k_{off} (1/s)	K_D (nM)	S_m
**3BNC117**	2G12 (n=2)	$9.7 \cdot 10^3$ $\pm 1.3 \cdot 10^2$	$1.4 \cdot 10^{-4}$ $\pm 3.9 \cdot 10^{-6}$	15 $\pm$ 0.60	2.7 $\pm 1.7 \cdot 10^{-2}$

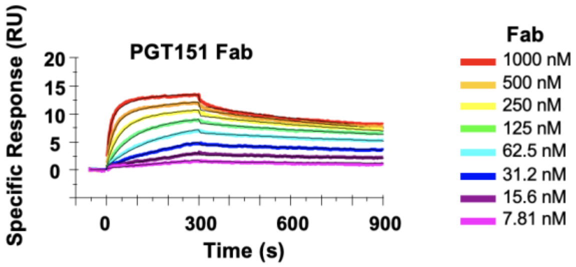

Heterogeneous-ligand modeling of PGT151 Fab binding to CZA97.012 SOSIP.664

Fab	Affinity purification	k_{on1} (1/Ms)	k_{off1} (1/s)	k_{on2} (1/Ms)	k_{off2} (1/s)	K_{D1} (nM)	K_{D2} (nM)	S_{m1}	S_{m2}	$S_{m1} + S_{m2}$
**PGT151**	2G12 (n=2)	$1.1 \cdot 10^5$ $\pm 1.4 \cdot 10^4$	$1.6 \cdot 10^{-4}$ $\pm 1.1 \cdot 10^{-4}$	$1.7 \cdot 10^4$ $\pm 3.3 \cdot 10^3$	$1.6 \cdot 10^{-3}$ $\pm 1.1 \cdot 10^{-5}$	1.6 $\pm$ 1.2	97 $\pm$ 18	0.66 $\pm 4.5 \cdot 10^{-2}$	0.56 $\pm 6.9 \cdot 10^{-2}$	1.2 $\pm$ 0

S4 Figure. PGT151 and 3BNC117 Fab binding to CZA97.012 SOSIP.664 analyzed with multi-cycle kinetics (MCK) by SPR. Fab of PGT151 or 3BNC117 at concentrations as indicated in the color-coded legend was injected over trimer immobilized freshly in each cycle. The trimer was 2G12-affinity- and then SEC-purified. Each sensorgram shows the response after background subtraction on the y axis in response units (RU) over time after start of injection on the x axis (s). Association was monitored for 300 s and dissociation for 600 s. The model-fitted functions (heterogeneous-ligand for PGT151 and Langmuir for 3BNC117) are shown as thin black curves. The number of replicate titrations and the fitted binding parameters are given in the tables underneath each sensorgram.
