# Supplementary material for: Glycan heterogeneity as a cause of the persistent fraction in HIV-1 neutralization
Source: PLoS Pathog. 2023 Oct 30;19(10):e1011601. doi: 10.1371/journal.ppat.1011601 (PMC10635575; doi:10.1371/journal.ppat.1011601)
Supplement: S5 Fig — (PDF) [file ppat.1011601.s008.pdf]

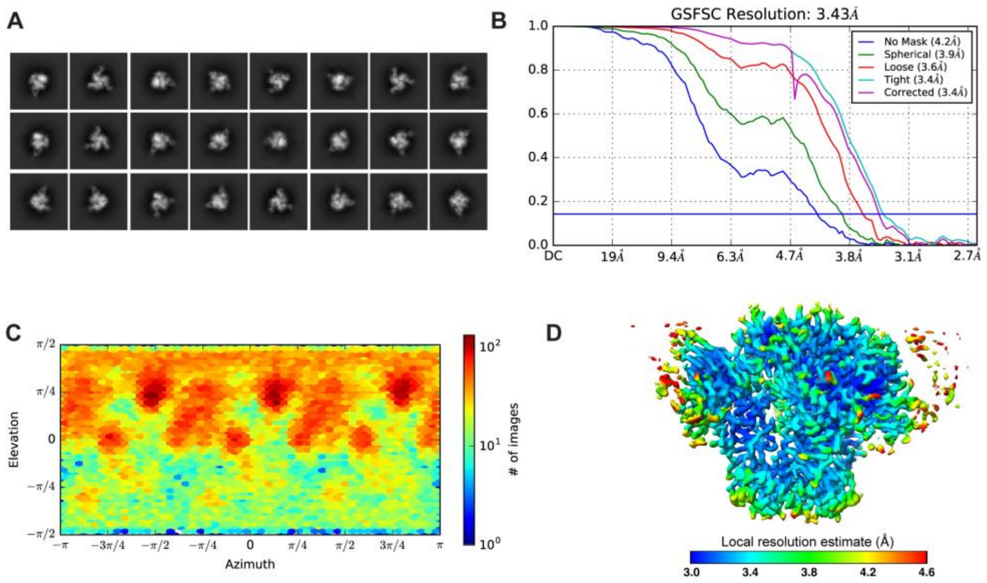

S5 Figure. Cryo-EM data processing. A. Representative 2D-class averages are shown. **B.** The diagram shows Fourier-shell correlation **C.** The angular distribution is given as depicted in the color code. **D.** Local resolution estimate (color-coded key) is shown for CZA97.012 SOSIP.664+3BNC117.
